# Long term outcome of Aldosteronism after target treatments

**DOI:** 10.1038/srep32103

**Published:** 2016-09-02

**Authors:** Vin-Cent Wu, Shuo-Meng Wang, Chia-Hui Chang, Ya-Hui Hu, Lian-Yu Lin, Yen-Hung Lin, Shih-Chieh Jeff Chueh, Likwang Chen, Kwan-Dun Wu

**Affiliations:** 1 Department of Internal Medicine, National Taiwan University Hospital, Taipei, Taiwan; 2 Department of Urology, National Taiwan University Hospital, Taipei, Taiwan; 3 Department of internal medicine, Taipei Tzu Chi Hospital, Buddhist Tzu Chi Medical Foundation, Taipei, Taiwan; 4 Cleveland Clinic Lerner College of Medicine and Glickman Urological and Kidney Institute, Cleveland Clinic, USA; 5 Institute of Population Health Sciences, National Health Research Institutes, No. 35, Keyan Road, Zhunan 350, Taiwan

## Abstract

There exists a great knowledge gap in terms of long-term effects of various surgical and pharmacological treatments on outcomes among primary aldosteronism (PA) patients. Using a validated algorithm, we extracted longitudinal data for all PA patients diagnosed in 1997–2010 and treated in the Taiwan National Health Insurance. We identified 3362 PA patients for whom the mean length of follow-up was 5.75 years. PA has higher major cardiovascular events (MACE) than essential hypertension (23.3% vs 19.3%, p = 0.015). Results from the Cox model suggest a strong effect of adrenalectomy on lowering mortality (HR = 0.23 with residual hypertension and 0.21 with resolved hypertension). While need for receptor antagonist (MRA) MRA after diagnosis suggests that a defined daily dose (DDD) of MRA between 12.5 and 50 mg may alleviate risk of death in a U-shape pattern. A specificity test identified patients who has aldosterone producing adenoma (HR = 0.50, p = 0.005) also confirmed adrenalectomy attenuated all-cause mortality. Adrenalectomy decreases long-term all-cause mortality independently from PA cure from hypertension. Prescription corresponding to a DDD between 12.5 and 50 mg may decrease mortality for patients needing MRA. It calls for more attention on early diagnosis, early treatment and prescription of appropriate dosage of MRA for PA patients.

Although the role of primary aldosteronism (PA) in increasing cardiovascular risk and the potential of targeted therapy for PA have gained recognition^1^,^2^, there exists a great knowledge gap in terms of long-term effects of various surgical and pharmacological treatments on outcomes among PA patients^3^. For instance, it remains unclear whether certain targeted treatments for PA can yield everlasting elimination of high blood pressure and regression of the adverse cardiovascular changes^4^,^5^.

A cohort study reported a higher risk of cardiovascular events in PA patients than in non-PA counterparts at diagnosis, and an indifferent risk level for PA patients in the follow–up after adrenalectomy or treatment with a mineralocorticoid receptor antagonist (MRA)^6^. While MRA treatment seems to produce a similar effect as adrenalectomy in terms of prevention of cardiovascular events^7^, it is unclear whether MRA treatment and adrenalectomy also grant a similar effect regarding all-cause mortality^3^.

A recent nationwide epidemiological study in Japan showed that surgical treatment had a larger effect of on ameliorating hypertension and hypokalemia than medical treatment^8^. While normalization of blood pressure and correction of hypokalemia are important, there are other goals for effectively managing PA and preventing organ complications^9^. It calls for more effort into unearthing better treatment patterns for reducing risk of mortality over a long term. Furthermore, it is worthwhile to investigate how adrenalectomy may lessen threat from death for PA patients with different statuses of subsequent of cardiovascular accidents (CVA), a well-recognized risk factor for all-cause mortality.

Claims databases comprehensively capturing information on episodes of care across healthcare settings could make disease outcomes research greatly more promising^10^. Taking advantage of the Taiwan National Health Insurance (NHI) research database, the aim of this study is to test PA patients receiving adrenalectomy would have a favorable probability of mortality over a long term than their counterpart patients without the operation. We further examined whether adrenalectomy would yield similar beneficial effects on decreasing all cause mortality between patients with subsequent CVA and those without CVA. We also conducted dose-response analysis regarding MRA use and mortality.

## Methods

### Data sources

The Taiwan NHI is a nationwide insurance program that covers outpatient visits, hospital admissions, prescriptions, intervention procedures and disease profiles for over 99% of the population in Taiwan (23.12 million in 2009). The NHI database is one of the largest and most comprehensive databases in the world, and has been offering research data in various studies on diagnoses, medication use, and hospitalizations^11^,^12^,^13^,^14^. This study extracted all of the patients with the diagnosis of PA form 23,725,083 beneficiaries in Taiwan. The NHI data is generally reliable, because the National Health Insurance Administration routinely audits claims data to prevent fraud in the NHI program^15^,^16^. Our study used a longitudinal database created by the National Health Research Institutes (NHRI) through extracting original NHI data for all patients who ever had PA diagnosis in the NHI in the period from 1997 to 2010.

### PA identification and mortality follow-up

**Figure 1:**
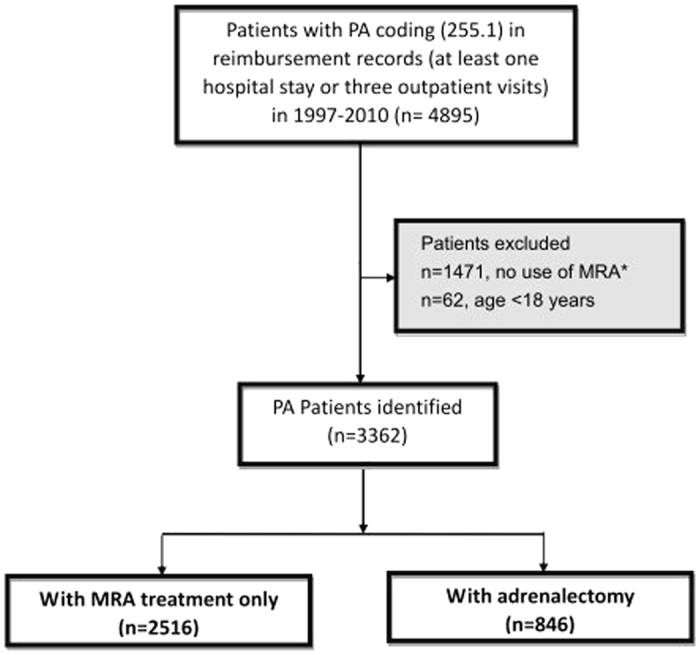
Flow diagram of selecting study subjects. (Abbreviations: MRA, mineralocorticoid receptor antagonist; PA, primary aldosteronism) *Our study only enrolled patients who ever used MRA (belonging to the ATC class C03D) in the one year prior to or the two years following the first ICD-9-CM coding of PA, because this additional condition could assure high values for both sensitivity and the positive predictive value according to our validated report^17^.

Our study used a validated algorithm to identify PA patients diagnosed in 1997–2010, and further enrolled PA patients aged ≥18 at the time of first medical record of PA (ICD code = 255.1). The administrative data on diagnosis and MRA prescription identified patients with primary aldosteronism in Taiwan had been validated^17^. Figure 1 depicts our procedures for selecting study subjects. Our study only enrolled patients who ever used MRA (belonging to the ATC class C03D) in the one year prior to or the two years following the first ICD-9-CM coding of PA, because this additional condition could assure high values for both sensitivity and the positive predictive value according to our validated report^17^. Two kinds of MRA drugs are listed in the guidelines for treating PA, and only one potassium sparing agent (spironolactone, ATC code = C03DA01) was available in Taiwan before 2011. We further separated PA patients into a group receiving adrenalectomy and another one receiving MRA treatment.

### Research variables

The demographic and clinical characteristics of all PA patients at their index diagnosis were recorded. The parameters included age, gender, the year of diagnosis, and various comorbid conditions, such as hypokalemia and hyperlipidemia. Each type of pre-diagnosis comorbidity was identified by a diagnosis history that indicated record for at least one hospital admission or at least three outpatient department visits during the one year immediately prior to the index diagnosis. This rule was constructed on the basis of a relatively strict criterion which was well validated^12^,^13^,^15^,^16^,^17^,^18^.

### Medication exposure and adrenalectomy

Each patient’s exposure to MRA (belonging to the ATC class C03D) was measured on the basis of the cumulative dose, and expressed as the defined daily dose (DDD) according to the definition by World Health Organization^19^. The DDD of MRA was calculated from 30 days to 365 days before mortality or the end of the study (December 31, 2010), whichever occurred first. We did not count medications prescribed within 30 days preceding any outcome event, in order to reduce potential confounding effects due to indication^16^,^20^. We categorized and recorded the administration of other antihypertensive pharmaceuticals at the time of PA diagnosis, and confirmed delivery of adrenalectomy by referring to NHI data on surgical procedures. We defined cure of hypertension by the operation as no demand for any antihypertensive medication for at least 1 year after adrenalectomy^20^,^21^. The NHI claims data regarding adrenalectomy and medications are reliable because they were constructed on the basis of NHI procedure and drug codes that were tied to NHI reimbursement system with auditing. The indication and guideline for hypertensive management in Taiwan has proposed and revised by the Taiwan society of Hypertension^22^. Briefly, A diagnostic algorithm was proposed, emphasizing the ESH/ESH joint hypertension guidelines suggestion to loosen BP targets to <140/90 mmHg for all patients^22^.

### Validation of the diagnostic procedures

Because there is no specific ICD diagnosis of aldosterone producing adenoma, for the comparison between patients with aldosterone producing adenoma who were treated with surgery vs MRA, PA patients with the diagnosis of adrenal tumor (ICD code = 227, 227.0, 239.7) were further analyzed as a specificity test. This tests included two medical centers (National Taiwan University Hospital (NTUH), Taipei, Taiwan; Taipei University Hospital, Taipei, Taiwan), and five regional hospitals (Cardinal Tien Hospital, New Taipei City, Taiwan; Taipei Tzu Chi Hospital, New Taipei City, Taiwan; Yun- Lin Branch of NTUH, Douliou City, Taiwan; Hsin-Chu Branch of NTUH, Hsin-Chu City, Taiwan; Zhongxing Branch of Taipei City Hospital, Taipei, Taiwan)^20^,^23^,^24^.

### Statistical analysis

Continuous variables are described as mean ±SD; discrete variables are presented as counts or percentages. We used the R software, version 2.8.1 (Free Software Foundation, Inc., Boston, MA, USA), and the Stata software, version 12 (StataCorp, College station, TX, USA). A two-sided p-value < 0.05 was considered statistically significant.

We drew Kaplan-Meier survival curves separately for patients receiving adrenalectomy and those without the operation, and conducted the log-rank test to compare risk of death between the two patient groups. As adrenalectomy, MRA prescription and potassium supplement (belonging to the ATC class A12B) for hypokalemia are main treatments following PA diagnosis, we used a Cox proportional hazards model with time-varying covariates to account for their influences on risk of death in our investigation of factors associated with long-term mortality among PA patients. Time-varying covariates took the value 0 before the start of treatments and could switch to 1 at the start of treatment. Each patient was followed from the date of the PA diagnosis to death or the end of the study (December 31, 2011), whichever occurred first.

On the basis of this hazard function, we further simulated and depicted 10-year survival curves of the probability of freedom from mortality under different scenarios of treatment in regard to MRA, and adrenalectomy among all APA patients^12^. Specifically, we stratified patients by the status of MACE after target treatments.

Clinical decisions for delivery of adrenalectomy are affected by patients’ clinical conditions. To assure the interval dataset validity of comparing adrenalectomy and non- adrenalectomy groups, we generated an alternative sample of PA patients using propensity score matching (PSM) process with the 1:1 matching ratio, and subsequently estimated an alternative Cox regression model. We constructed a propensity score using a non-parsimonious multivariable logistic regression model in an attempt to make an unbiased estimate of the indicators predicting adrenalectomy. The predicted probability derived from the logistic equation was used as the propensity score for each individual.

To determine threshold values of MRA dosage for favorable survival chances, we specified a multilevel discrete-time event history analysis utilizing the logistic regression method incorporating patient-specific random effects, and adopted a generalized additive model (GAM) with adjustment for risk factors listed in Table 1 and with splines regarding MRA dosage. This approach permits adjustments for possible nonlinear effects of continuous variables^25^,^26^. To show the effect of MRA dosage on risk of death, we plotted a function curve with values of the logs of odds ratios. The curve was centered to have an average of zero over the range of the data. The approximate point-wise 95% CIs were also depicted. With dosage threshold values determined, we categorized MRA dosage levels accordingly, and incorporated the non-linear effect of MRA dosage on risk of death into the specification of another alternative Cox regression model for long-term mortality to further examine the non-linear effect on long-term mortality.

### Ethical considerations

Because patients were anonymous in our study, no informed consent was required. As the identification numbers of all individuals in the NHI research databases were encrypted to protect the privacy of the individuals, this study was exempt from a full ethical review by the institutional review board of National Taiwan University Hospital (201303017RINC). All of the methods were carried out in “accordance” with the approved guidelines.

## Results

### Patient characteristics

Among the 4895 patients with PA diagnosis in 1997–2010, we identified 3362 PA patients (68.7%) based on our diagnosis algorithm (Fig. 1), and diagnostic procedures from national health insurance data was listed in stable 1. The mean age was 51.3 ± 14.7 years at PA diagnosis, and 46.3% of them (n = 1557) were male. During the follow up, 846 patients (25.2%) underwent adrenalectomy. Table 1 summarizes the demographic and clinical characteristics of PA patients by their status of receiving adrenalectomy.

We found no significant difference in the incidence rate of coronary artery disease between the two groups at the baseline. Patients without adrenalectomy had higher incidence rates of congestive heart failure, cerebrovascular disease, chronic kidney disease (CKD), chronic obstructive pulmonary disease (COPD), diabetes mellitus (DM), moderate or severe liver disease, and several other morbid conditions. The non-adrenalectomy group also had a larger proportion of taking diuretics. In contrast, patients with the operation had higher risk of hypokalemia, and tended to have higher demand for potassium supplement, calcium channel blockers, angiotensin converting enzyme inhibitors (ACEI), and angiotensin receptor blockers (ARB). In 846 patients receiving adrenalectomy, 652 (77.1%) had no demand for any antihypertensive drug for at least one month immediately following the operation, and 517 (61.1%) demanded none for at least one year immediately post the surgery.

### All- cause mortality

#### All enrollee

After a mean follow-up of 5.75 years, 452 patients (13.4%) died. The incidence rate of death was 23.4 per 1000 person-years. PA patients with adrenalectomy had a lower all-cause mortality than those without the operation (3.8% v.s. 16.7%, p < 0.001). The group with the operation also had a lower incidence rate of cardiovascular event (19.3% v.s. 23.3%, p = 0.015).

A detailed analysis of hospital care immediately before death indicates that 42.0% of all deaths were attributable to cardiovascular disease (Table S2). Comparison between survivors and non-survivors (Table S2) shows that survivors had a lower proportion of men (44.7% v.s. 56.9%, p < 0.001), and were younger than non-survivors (49.6 ± 13.7 v.s. 62.7 ± 15.7, p < 0.001). The premorbid risks were more severe in non-survivors (all p-values < 0.05), except rheumatologic disease. Survivors tended to have lower proportion of calcium channel blockers, diuretics, and ACEI or ARB at PA diagnosis (all p-values < 0.05). Survivors also tended to have lower demand for potassium supplement after PA diagnosis (42.2% v.s. 63.5%, p < 0.001). In contrast, survivors were more likely to undergo adrenalectomy (28.0% v.s. 7.1%, p < 0.001). Kaplan-Meier curves of freedom from mortality for PA patients receiving adrenalectomy and those without operation also depicts that the group with adrenalectomy had favorable survival advance (p < 0.001 for the log-rank test, Fig. S1).

For all-cause mortality, our Cox proportional hazards model with time-varying covariates had a good validity (C-index = 0.83; Table 2). Adrenalectomy appeared to be very protective. The protective effect was independent of effects from age (hazard ratio (HR) = 1.05 for an increase by a year), gender (HR = 1.31 for male), CKD (HR = 2.16), congestive heart failure (HR = 1.90), Liver disease (HR = 1.87), coronary artery disease (HR = 1.90), dementia (HR = 1.73), hemiplegia (HR = 2.68), DM (HR = 1.42), and solid tumor (HR = 1.81) (all p-values < 0.001). Either operation leading to cure of hypertension (HR = 0.25, p < 0.001) or that leaving residual hypertension (HR = 0.28, p < 0.001) were protective. Need for MRA after diagnosis did not signal an impact on mortality (p = 0.237), and so did demand for potassium supplement before operation(HR = 1.94, p < 0.001).

#### Patients matched by propensity score

Because the group treated medically was sicker in terms of several chronic disease by univariate analysis at disease diagnosis, propensity score matched (PSM) was performed to balance the comorbidities. Our logistic regression model of propensity score identified several factors predicting adrenalectomy (p = 0.651 for the Hosmer-Lemeshow goodness of fit test; Table S3). After the PSM process, we found matches for 822 PA patients (97.2%) receiving adrenalectomy. Comparison between these 822 PA patients and their matching counterparts (Table 1) indicates adequate comparability between the two groups. The two groups had similar characteristics in gender, age, premorbid risk, and antihypertensive drugs used at diagnosis, although patients with the operation appeared to have a larger demand for potassium supplement before operation than their matching counterparts. Regarding outcomes, we found PA patients with adrenalectomy had a lower all-cause mortality than their matching counterparts (3.8% v.s. 11.7%, p < 0.001).

Results from the alternative Cox proportional hazards model using the sample generated after the PSM process (C-index = 0.79; Table 2) also indicate a very protective effect of adrenalectomy, with (HR = 0.23, p < 0.001) or without residual hypertension (HR = 0.21, p < 0.001). Need for potassium supplement after PA diagnosis still signaled a higher mortality. In contrast, although the point estimate of the HR regarding the association of need for MRA after PA diagnosis with mortality still suggests a link between need for MRA and higher risk of death, its large variance hints that the use of MRA might affect the level of risk from death. This observation led us to further conduct a dose-response analysis regarding MRA use.

#### Comparison of the effect of adrenalectomy on long-term risk of mortality under a framework of subgroup analysis

**Figure 2:**
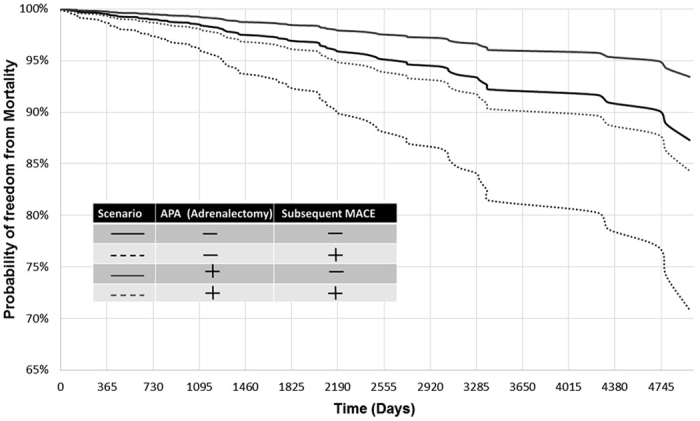
Future 10-year probability of mortality was lower among APA patients received adrenalectomy during follow up. The simulation curves were depicted based on different scenarios of morbid conditions with regard to MRA, and adrenalectomy stratified by subsequent MACE.

To investigate the consistency in the beneficial effect of adrenalectomy among different groups in PA patients, we further conducted subgroup analysis with respect to baseline comorbidity that further adjusted for age and gender. We found that adrenalectomy was consistently associated a much lower long-term risk of death across broad varieties of patient groups (Fig. 2).

#### The diagnosis of adenoma and specificity analysis

In our dataset, among the patients who underwent adrenalectomy and had the ICD-9 record of an adrenal tumor, there was a very high positive predictive value (96%) of APA. Furthermore, in the specificity test of this study we chose- only to include those confirmed APA patients to run the test, which although sacrificed some sensitivity, increased the positive predictive rate. In such a conservative way, we are confident to report the beneficial effects of adrenalectomy on the all-cause mortality among these APA patients.

**Figure 3:**
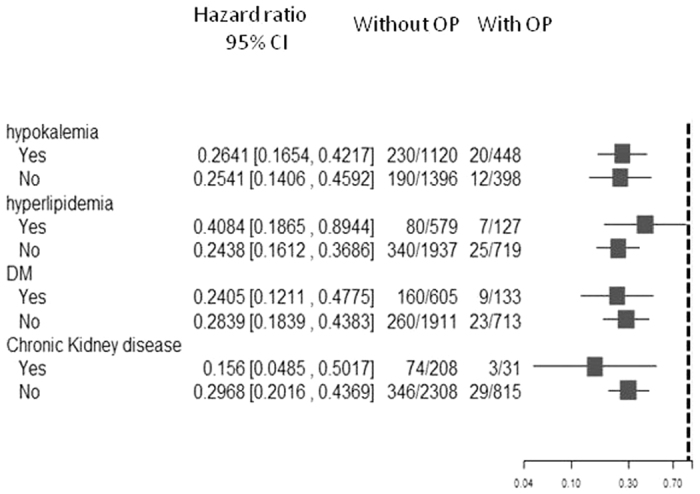
Adjusted HRs for long-term risk of mortality among PA patients, based on comparison between adrenalectomy and non-adrenalectomy groups, and subgroup analysis with respect to premorbid risk that further adjusted for age and gender. (Abbreviations: CI, confidence interval; DM, diabetes mellitus; HR, hazard ratio; OP, operation; PA, primary aldosteronism).

We further focus on patients with aldosterone producing adenoma as a patient with adenoma may be treated either with unilateral adrenalectomy or with MRA. The Cox proportional hazards model using aldosteronism patients with the diagnosis of adrenal tumor indicate a protective effect of adrenalectomy (HR = 0.50, p = 0.005) and non-significant effect of MRA prescription (HR = 1.72, p = 0.051). Need for potassium supplement after PA diagnosis still signaled a higher mortality (HR = 2.65, p = 0.005). In APA patients, our simulation results (Fig. 3) showed non-adrenaectomy had an ominous long-term association with the risk of mortality compared with a reference APA patient with adrenalectomy and without subsequent MACE for the whole period of 10 years after discharge. Subsequent MACE will augment the risk effect of non-adrenalectmoy on survival.

Liver dysfunctions such as liver cirrhosis could induce secondary aldosteronism involving hypokalemia which often require spironolactone. We do subgroup analysis enrolling patients without liver disease. In line with the main result, adrenalectomy showed protective role (HR, 0.27, p < 0.01), however received MRA did not (HR, 0.93, p = 0.61).

#### Dose-response analysis regarding MRA use and mortality

**Figure 4:**
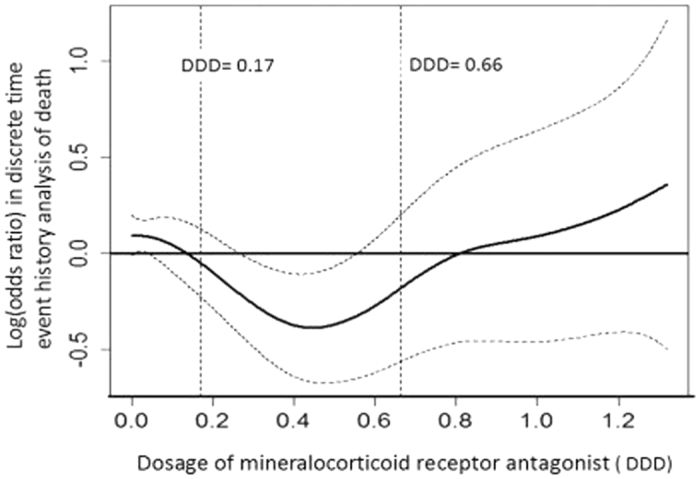
The function curve with values of the logs of odds ratios from the GAM model with splines regarding MRA for our multilevel discrete-time event history analysis of risk of death among PA patients. The curve was centered to have an average of zero over the range of the data. The dashed lines indicated approximated point-wise 95% CIs. (Abbreviations: CI, confidence interval; DDD, defined daily dose; GAM, generalized additive model; MRA, mineralocorticoid receptor antagonist; PA, primary aldosteronism) (The DDD of MRA is 0.17 (12.5 mg of spironolactone) and 0.66 (50 mg spironolactone).

Results from our GAM model indicate two threshold values for the dosage of MRA regarding long-term mortality appeared in a U shape pattern: DDD = 0.17 (12.5 mg) and 0.66 (50 mg) (Fig. 4). Results from the Cox regression model incorporating the non-linear effect of MRA dosage on risk of death show that the adjusted HR of DDD below 0.08 relative to DDD between 0.17 and 0.66 was 1.52 (p = 0.001), and that of DDD larger than 0.66 was 1.57 (p = 0.044) (Table 3 and S4). These results suggest either underuse or overuse of MRA might lead to a higher mortality. Results from our additional analysis of the numbers of death incidences and the numbers of person-years in our follow-up show that few PA patients were exposed to DDD larger than 0.66 (dose equal to 50 mg of spironolactone); in contrast, a large proportion of PA patients were exposed to DDD lower than 0.17 (dose equal to 12.5 mg of spironolactone) (Table 3).

## Discussion

Our research provides the first empirical evidence from a large population cohort, crossing the entire disease spectrum and representative of the whole patient population that adrenalectomy may reduce long-term all-cause mortality among PA patients, while MRA prescription may also help in this regard when its dosage is appropriate. These findings suggest a more beneficial effect of adrenalectomy over medication on long-term all-cause mortality among PA patients, particularly in those suitable for the operation.

Our finding that cardiovascular event is the leading cause of mortality in PA is consistent with this interpretation. High aldosterone levels are also associated with mortality in heart failure cohorts^27^. Our comparison of long-term risk of all-cause mortality among patient groups stratified by status of MACE and adrenalectomy further suggests that high aldosterone levels may be as fatal as subsequent MACE, and deserves more attention in mortality reduction. Our comparison of the effect of adrenalectomy on long-term risk of mortality under a framework of subgroup analysis shows that adrenalectomy could ameliorate all-cause mortality and is independent of other co-morbidities including DM. This highlights the potential value of preventing mortality by efficiently controlling aldosterone level in PA patients, and well taking the advantage of target treatment.

### Adrenalectomy and MRA

The aforementioned Japanese nationwide epidemiological study reported a better effect of surgical treatment than pharmacological treatment on improving hypertension and hypokalemia^28^. Previous publication in this field have also shown a difference in the cost-benefit between patients receiving adrenalectomy and those only treated with MRA^29^. There are differential impact of adrenalectomy and MRA in regard to ventricular mass regression, with fewer or no effect of MRA on left ventrical index^21^,^30^,^31^. Our study furthers knowledge in this field by demonstrating a difference in long term-mortality between these two patient groups. The comparison of adrenalectomy with MRA in aldosterone producing adenoma or in patients who do not have liver disease identified in our specificity test also showed consistent result. Aldosterone-producing adenoma is usually amenable to unilateral adrenalectomy, or pharmacological approaches using MRA.

In the case of subsequent MACE (Fig. 3), the mortality rate will further increase. APA patients who did not receive adrenalectomy, is almost equally to subsequent MACE leading to all-cause mortality. Our result showed adrenalectomy attenuated all cause of mortality among APAs and also raised on the possibility of adrenalectomy to be on target treatment for APA patients. These findings are consistent with a report of the German Cohort^3^, which was resulted from only univiariate analysis.

Our study also reveals the importance of dose-response analysis regarding MRA use. The non-monotonic direction of the association between the cumulative defined dose and the mortality rate deserves attention. Both inadequate dosage of MRA (lower than DDD = 12.5 mg, as suggested by our analysis) and the extreme high dosage (larger than DDD = 50 mg), which is related to difficult disease control, are linked with higher long-term mortality. It is found that modest levels of MRA are sufficient to control not only BP but also cardiovascular damage in both PA patients, even at these modest doses (average at 3 years, 29 mg/day), renal function was considerably improved^32^. These phenomena suggest that underuse of MRA at clinical practice might be a problem calling for more attention.

An important fact is that research consistently determines larger reduction in blood pressure (BP) and hyperaldosteronism after adrenalectomy and that could attribute to detrimental genomic and nongenomic effects^33^. Also noteworthy is the finding that adrenalectomy might yield a therapeutic effect more rapidly, which was shown to ameliorate cardiac hypertrophy in PA patients^6^. In contrast, findings regarding benefits of MRA use are limited, probably partly due to frequent occurrence of dose-dependent side-effects, especially gynecomastia, and subsequent decrease in efficacy^34^. MRA treatment may also induce an increase in aldosterone, and subsequently trigger a vicious cycle with nongenomic effects that leads to an insufficient effect of prescribed MRA on blocking mineralocorticoid receptor activated by high plasma aldosterone level^35^.

There are other concerns about MRA prescription, such as medication compliance. As shown in an Italy study, MRA treatment was discontinued in 28% of PA patients^36^. Possible delays in realization of the effects of MRA on improving renal function, lowering blood pressure and ameliorating indices in artery stiffness are among the concerns^35^,^37^. As demonstrated by our prospective study, proteinuria and cystatin C, a marker of kidney function, dropped off soon after adrenalectomy, but still failed to significantly decrease one year after initiation of MRA treatment^37^. Also noteworthy is the finding that adrenalectomy might yield a therapeutic effect more rapidly^6^, and it takes more than one year to ameliorate overhydration^38^ and 6 years to observe reductions in left ventricular wall thickness and mass in PA patients after MRA treatment^21^. Most of these studies have demonstrated that medically treated patients require much longer-term follow-up to manage their condition, whereas most surgical patients can be successfully discharged shortly after surgery. When possible, surgical management may represent a more expeditious means of treating PA. In summary, correction of hyperaldosterone and an appropriate dosage of MRA, as well as an adequate duration of medication, are expected to abate harms from adverse events caused by aldosteronism.

### Restoration of normotension

Around one third of our patient cohort still demanded hypertensive medication one year after adrenalectomy, which is in accordance with previous observations^20^,^27^. Nevertheless, our study found that adrenalectomized patients would have lower long-term risk of death, regardless of whether they had resolved hypertension that needs no antihypertensive medication or residual hypertension that still requires pharmaceutical treatment. Vascular remodeling is less reversible, and difficulty in curing hypertension increases along with increase in the length of time under aldosterone exposure, independent of the normalized level of serum aldosterone^39^. Related to the phenomena, medically treated PA patients tend to have a larger need for antihypertensive treatment along with the passage of time, in order to achieve BP control^28^. A prospective study investigating on blood pressure demonstrated that the adrenalectomized patients required significantly less drugs than medically treated^30^. Japanese patients with APA, surgical treatment was significantly associated with amelioration of hypertension, while medical treatment showed no relationship^8^.

Consistent with our finding on a positive relationship between the age at diagnosis and the level of long-term mortality, an older age at diagnosis is associated with a higher likelihood of postoperative residual hypertension^20^,^23^ and cardiovascular events^30^. Among patients with resistant hypertension, 5–13% have PA^40^,^41^. An important implication on clinical practice from these findings is that early diagnosis and early treatment of PA are essential for well controlling BP with tapering antihypertensive medications. In addition to underscoring the importance of a timely identification of PA to accomplish regression of BP and reductions in cardiovascular risk and long-term all-cause mortality, our study highlights the benefits of targeted surgery and appropriate medication. Particularly, adrenalectomy could bring forth a striking healing effect, and appears to be a highly promising treatment strategy.

Our results mean that adrenalectomy decreases all cause mortality independently from PA cure and MRA will increase all-cause mortality in PA patients. A previous study showed no difference between surgically and medical treated patients with PA in terms of incidence of cerebral vascular accident^30^. Difficulties in diagnosing PA unequivocally, inadequate matching among patients, and insufficient statistical power for rare events, along with differences in disease severity and duration of hypertension at diagnosis, can account for discrepancies in findings from different studies^30^. Further randomized trials are warranted to confirm the large beneficial effect of adrenalectomy on mortality in observational studies.

### Study strengths and limitations

The insurance disease name could not represent the severity of the disease, which includes variety of clinical status. Our research database does not contain several unmeasured confounders, including body mass index, smoking status, blood pressure, potassium and aldosterone level, which are associated with long-term mortality. Furthermore, we could not identify PA patients with bilateral adenoma or unilateral hyperplasia and thus the difference between them was obscure. However the incidence of the two subgroups is relative low^42^ and our results were robust across the different models, strengthening our conclusion that. In contrast, our study has some strengths. The NHI database has a large national sample size and a long follow-up. Reliability of ICD-9-CM codes for PA and its co-morbidities were detailed validated in our large cohort^17^. Furthermore, our analysis taking into account time-varying covariates and dose-response regarding MRA can enrich the scope of research in this field. Time varying factors, including using MRA, cumulated dose of MRA, and K supply as time-dependent covariate in the model, ensured that patients were at risk only when they have used them. As long as patients were not receiving time varying treatments, they contributed to the untreated group and acted as controls for the treated patients during the treatment period and thus attenuated the confounders by indications. The model allowed patients to switch from one exposure group to countermanding period. In the repeated analysis, person days of follow-up were correctly classified as untreated until the intended treatment definition of “each immortal time of use” was met, and as treated thereafter^43^. We only shows a minimal difference in cardiovascular events between PA patients treated by adrenalectomy (19%) or MRA(23%). Although we have chosen the diagnosis time as starting time for survival analysis, the delay between diagnosis and adrenalectomy could be the healthy survivor bias. If patients with adrenalectomy are more often followed–up in medical care than patients with MRA co-intervention and proficiency biases are likely.

A noteworthy statement we would like to make is that the data retrieved and examined in this article were from health insurance registration data, where the diagnostic tests were not ‘controlled’ as ideal as some single or multi-center clinical studies. Nevertheless, imperfect as we admit it is, it is still the most ‘down-to-earth’ ‘real-life’ data that we could get to allow us to scrutinize some large-scale long-term outcomes of the PA patients among a population of 23 million people, according to the ICD-9 and CPT coding. We consider this ‘big-data’ approach offers several precious results and insights into the understanding of PA that were not examined and could not be offered via the smaller-scale multicenter studies. We have added the above paragraph into our discussion of the limitation of the study section.

However, a placebo-controlled randomized clinical trial, regard to MRA or adrenalectomy over as many years as in our observational study is not possible nor is it ethically acceptable to conduct; our prospective follow-up study provided a valid alternative^44^.

#### Prospective

Our result showed patients treated medically may be commonly under dosed is an important finding that would add to the medical literature for on target treatment of aldosteronism. It also reinforces the argument that early surgery in PA patients with confirmed adrenal lateralization should not be delayed in lieu of serial observation aimed at maximizing medical therapy, as the deleterious effect of elevated aldosterone on the cardiovascular system may be irreversible. The wide inter-patient variability of the disease probably needs individually tailored intervention.

## Conclusions

Adrenalectomy decreases long-term all-cause mortality independently from PA cure from hypertension. The effect of PA who did not receive adrenalectomy to all-cause mortality is similar to subsequent MACE. Among patients with need for MRA after PA diagnosis, prescription corresponding to a DDD between 12.5 and 50 mg may decrease mortality. Our study generated the largest study cohort of PA patients in the world currently, and provides the first in-depth analysis on the beneficial effects of adrenalectomy and appropriate dosage of MRA prescription on survival benefit. It calls for more attention on early diagnosis, early treatment and prescription of appropriate dosage of MRA for PA patients.

## Additional Information

**How to cite this article**: Wu, V.-C. *et al*. Long term outcome of Aldosteronism after target treatments. *Sci. Rep.* **6**, 32103; doi: 10.1038/srep32103 (2016).

## Supplementary Material

Supplementary Information

## Figures and Tables

**Table 1:** Comparison of characteristics between PA patients receiving adrenalectomy and those without operation.

	Before Match		*p*	After Match*		*p*
	No Operation (n = 2516)	Operation (n = 846)		No operation (n = 822)	Operation (n = 822)	
Male gender	1188 (47.2%)	369 (43.6%)	0.073	358 (43.6%)	360 (43.8%)	0.960
Age (in year)	52.91 ± 15.44	46.6 ± 10.85	<0.001	46.9 ± 13.7	46.9 ± 10.8	0.447
*Premorbid risk*						
Cushing syndrome	25 (1.0%)	2 (0.2%)	0.042	7 (0.9%)	2 (0.2%)	0.178
Hypothyroidism	20 (0.8%)	1 (0.1%)	0.039	6 (0.7%)	1 (0.1%)	0.124
Hyperthyroidism	53 (2.1%)	18 (2.1%)	0.999	22 (2.7%)	18 (2.2%)	0.632
Congestive heart failure	225 (8.9%)	32 (3.8%)	<0.001	48 (5.8%)	32 (3.9%)	0.085
Cerebrovascular disease	355 (14.1%)	88 (10.4%)	0.006	110 (13.4%)	88 (10.7%)	0.111
CKD	208 (8.3%)	31 (3.7%)	<0.001	25 (3%)	31 (3.8%)	0.497
COPD	309 (12.3%)	46 (5.4%)	<0.001	46 (5.6%)	46 (5.6%)	0.999
Coronary artery disease	57 (2.3%)	15 (1.8%)	0.492	10 (1.2%)	15 (1.8%)	0.421
Dementia	57 (2.3%)	2 (0.2%)	<0.001	1 (0.1%)	2 (0.2%)	0.999
Diabetes Mellitus	605 (24%)	133 (15.7%)	<0.001	140 (17%)	131 (15.9%)	0.595
Gout	247 (9.8%)	69 (8.2%)	0.173	82 (10%)	69 (8.4%)	0.305
Hemiplegia	30 (1.2%)	7 (0.8%)	0.45	9 (1.1%)	7 (0.9%)	0.803
Hyperlipidemia	579 (23%)	127 (15%)	<0.001	141 (17.2%)	126 (15.3%)	0.349
Hypokalemia	1120 (44.5%)	448 (53%)	<0.001	404 (49.1%)	432 (52.6%)	0.183
Moderate or Severe liver disease	243 (9.7%)	61 (7.2%)	<0.001	83 (10.1%)	60 (7.3%)	0.054
Peptic Ulcer	508 (20.2%)	116 (13.7%)	<0.001	5 (0.6%)	10 (1.2%)	0.300
Peripheral vascular disease	28 (1.1%)	10 (1.2%)	0.852	46 (5.6%)	46 (5.6%)	0.999
Rheumatologic disease	34 (1.4%)	10 (1.2%)	0.861	47 (5.7%)	47 (5.7%)	0.999
Solid tumor	134 (5.3%)	47 (5.6%)	0.792	110 (13.4%)	88 (10.7%)	0.111
Antihypertensive drugs used at diagnosis						
α- blockers	319 (12.7%)	120 (14.2%)	0.263	112 (13.6%)	119 (14.5%)	0.670
β- blockers	924 (36.7%)	333 (39.4%)	0.175	331 (40.3%)	321 (39.1%)	0.650
Calcium channel blockers	1476 (58.7%)	578 (68.3%)	<0.001	552 (67.2%)	555 (67.5%)	0.916
Diuretics	547 (21.7%)	112 (13.2%)	<0.001	95 (11.6%)	111 (13.5%)	0.264
ACEI/ARB	960 (38.2%)	405 (47.9%)	<0.001	371 (45.1%)	386 (47.0%)	0.488
K supply after PA diagnosis	1026 (40.8%)	490 (57.9%)	<0.001	373 (45.4%)	478 (58.2%)	<0.001
Antihypertensive drugs used at event						
α- blockers	104 (4.1%)	13 (1.5%)	<0.001	35 (4.3%)	13 (1.6%)	0.002
β- blockers	502 (20%)	109 (12.9%)	<0.001	162 (19.7%)	108 (13.1%)	<0.001
Calcium channel blockers	685 (27.2%)	126 (14.9%)	<0.001	200 (24.3%)	125 (15.2%)	<0.001
Diuretics	367 (14.6%)	61 (7.2%)	<0.001	102 (12.4%)	61 (7.4%)	0.001
ACEI/ARB	515 (20.5%)	125 (14.8%)	<0.001	153 (18.6%)	125 (15.2%)	0.076
Outcomes						
Cardiovascular event	587 (23.3%)	163 (19.3%)	0.015	176 (21.4%)	154 (18.7%)	0.048
All-cause mortality	420 (16.7%)	32 (3.8%)	<0.001	96 (11.7%)	31 (3.8%)	<0.001

123123.

Abbreviations: ACEI, angiotensin converting enzyme inhibitor; ARB, angiotensin receptor blocker; CKD, chronic kidney disease; COPD, chronic obstructive pulmonary disease; K, potassium; PA, primary aldosteronism.

^*^After the propensity score matching process, only 822 PA patients could have their matching counterparts.

**Table 2:** Factors associated with long-term mortality in a Cox regression model* taking into account time-varying covariates.

	Before match*			*p*	After match**			*P*
	HR	Lower 95% CI	Upper 95% CI		HR	Lower 95% CI	Upper 95% CI	
Age (in year)	1.05	1.04	1.06	<0.001	1.05	1.03	1.06	<0.001
Male gender	1.31	1.08	1.59	<0.001				
Premorbid risk								
CKD	2.16	1.62	2.87	<0.001				
Congestive heart failure	1.90	1.47	2.46	<0.001				
Liver disease	1.87	1.44	2.42	<0.001	2.13	1.35	3.35	<0.001
Coronary artery disease	1.90	1.30	2.77	<0.001				
Dementia	1.73	1.09	2.74	<0.019				
Hemiplegia	2.68	1.68	4.28	<0.001	3.77	1.81	7.89	<0.001
Diabetes Mellitus	1.42	1.16	1.73	<0.001				
Solid tumor	1.81	1.35	2.43	<0.001	3.39	2.15	5.37	<0.001
Time-varying covariate								
Adrenalectomy								
Residual hypertension+	0.28	0.18	0.44	<0.001	0.23	0.13	0.26	<0.001
Resolved hypertension+	0.25	0.14	0.44	<0.001	0.21	0.10	0.24	<0.001
MRA	1.15	0.91	1.45	0.237	1.53	0.19	2.59	0.110
K supply	1.94	1.47	2.56	<0.001	1.64	1.06	2.55	0.028

Abbreviations: CI, confidence interval; CKD, chronic kidney disease; COPD, chronic obstructive pulmonary disease; HR, hazard ratio; K, potassium; MRA, mineralocorticoid receptor antagonist.

^*^The final model had a good validity (C-index = 0.83).

^**^The final model had a good validity (C-index = 0.79).

^+^Cure of hypertension is defined as no use of antihypertensive drug for at least 1 year after adrenalectomy.

**Table 3:** Dose-response analysis regarding MRA use and mortality among PA patients.

	Time varying number at mortality or censer-points	Incidence rate (per 1,000 person-years)	Adjusted HR (95%CI )	*P*
Daily dose of MRA (expressed as DDD/ Dose)*				
<0.17 (12.5 mg)	358	22.67	1.52 (1.18–1.96)	0.001
0.17–0.66 (12.5–50 mg)	196	19.40	1 (ref.)	
>0.66 (50 mg)	59	17.47	1.57 (1.01–2.44)	0.044

Abbreviations: CI, confidence interval; DDD, defined daily dose; HR, hazard ratio; MRA, mineralocorticoid receptor antagonist; PA, primary aldosteronism.

^*^The DDD of MRA was calculated form 30 days to 365 days before mortality or the end of the study.
