# Corrigendum: Long term outcome of Aldosteronism after target treatments

**DOI:** 10.1038/srep45249

**Published:** 2017-03-24

**Authors:** Vin-Cent Wu, Shuo-Meng Wang, Chia-Hui Chang, Ya-Hui Hu, Lian-Yu Lin, Yen-Hung Lin, Shih-Chieh Jeff Chueh, Likwang Chen, Kwan-Dun Wu

Scientific Reports 6: Article number: 3210310.1038/srep32103; published online: 09 02 2016; updated: 03 24 2017

The original version of this Article contained an error in the Abstract.

“While need for receptor antagonist (MRA) MRA after diagnosis suggests that a defined daily dose (DDD) of MRA between 12.5 and 50 mg may alleviate risk of death in a U-shape pattern”.

now reads:

“While the need for mineralocorticoid receptor antagonist (MRA) after diagnosis suggests that a defined daily dose (DDD) of MRA between 12.5 and 50 mg may alleviate risk of death in a U-shape pattern”.

This error has now been corrected in the PDF and HTML versions of the Article.

