# Supplementary material for: Long term outcome of Aldosteronism after target treatments
Source: Sci Rep. 2016 Sep 2;6:32103. doi: 10.1038/srep32103 (PMC5009379; doi:10.1038/srep32103)
Supplement: Supplementary Information [file srep32103-s1.doc]

Long term outcome of Aldosteronism after target treatments: 3362 patients from Taiwan nationwide longitudinal cohort-based study

**Authors:** Vin-Cent Wu, Shuo-Meng Wang, Chia-Hui Chang, Ya-Hui Hu, Lian-Yu Lin, Yen-Hung Lin, [Shih-Chieh Jeff Chueh](http://jra.sagepub.com/search?author1=Shih-Chieh+Jeff+Chueh&sortspec=date&submit=Submit), Likwang Chen, Kwan-Dun Wu

**Supplementary files**

**Material and methods**

**The rate and accuracy of diagnostic procedures of PA in Taiwan.**

In Taiwan, patients who received captopril tests showed a sensitivity of 66.2% and a specificity of 89.1% ^1^. According to our previous validation, the NP-59 for Primary Aldosteronism patients showed an accuracy of 77.4% for predicting pathological change of aldosteronism^2^; However NP-59 semi-quantification in differentiating APA could reach the accuracy of 85.7%^3^.

During the period from 1997-2010, the overall success rate of Dyna CT-assisted adrenal venous sampling (AVS) in TAIPAI group was 87%, including an individual success rate of 88% for the right side adrenal tumor and 97% for the left side adrenal tumor. The overall procedural time was 49.5 ± 21.3 min^4^.

**Validation of APA form TAIPAI database**

This validation included two medical centers (National Taiwan University Hospital (NTUH), Taipei, Taiwan; Taipei University Hospital , Taipei, Taiwan), and five regional hospitals (Cardinal Tien Hospital, New Taipei City, Taiwan; Taipei Tzu Chi Hospital, New Taipei City, Taiwan; Yun- Lin Branch of NTUH, Douliou City, Taiwan; Hsin-Chu Branch of NTUH, Hsin-Chu City, Taiwan; Zhongxing Branch of Taipei City Hospital, Taipei, Taiwan). ^5,6^.

.

**Results**

***The details and validation of diagnostic procedures (table s3 and table s4)***

CT scan, postural stimulation and AVS were the leading procedures to identify APA from our two datasets (the NHI dataset of the current article and the TAIPAI dataset). Salt loading test is the most commonly used diagnostic procedure to identify PA form the TAIPAI database. For the comparison between patients with aldosterone producing adenoma who were treated with surgery versus MRA, PA patients with the diagnosis of adrenal tumor (ICD-9 code =227, 227.0, 239.7) were further analyzed as a specificity test from TAIPAI cohort. In our dataset, among the patients who underwent adrenalectomy and had the ICD-9 record of an adrenal tumor, there was a very high positive predictive value (96%) of APA. Furthermore, in the specificity test of this study we chose- only to include those confirmed APA patients to run the test, which although sacrificed some sensitivity, increased the positive predictive rate. In such a conservative way, we are confident to report the beneficial effects of adrenalectomy on the all-cause mortality among these APA patients.

Among the patients who underwent AVS, we further identified those patients with incidentaloma (not APA) and contralateral adrenal aldosteronism, and compared them with the previously mentioned combo coding of PA and adrenal tumor. The result showed a high incidence of such discrepancy (99.2%) and indirectly confirmed the high specificity of coding about adrenal adenoma among PA patients as APA.

**Supplementary Tables**

**Table S1.**

***The details of diagnostic procedures* from national health insurance data**

|  | ***Before Match*** | |  | ***After Match*** | | |  |
| --- | --- | --- | --- | --- | --- | --- | --- |
|  | **No operation**  **(n=2516)** | **Operation**  **(n=846)** | ***p*** | **No operation (n=822)** | **Operation (n=822)** | ***p*** |  |
| **Male gender** | 1188 (47.2%) | 369 (43.6%) | 0.073 | 358 (43.6%) | 360 (43.8%) | 0.960 |  |
| **Age (in year)** | 52.91 ± 15.44 | 46.6 ± 10.85 | <0.001 | 46.9± 13.7 | 46.9 ± 10.8 | 0.447 |  |
| **Age < 35 y/o** | 292 (11.6%) | 114(13.5%) | 0.161 | 140(17.0%) | 107(13.0%) | 0.027 |  |
| **Age < 40 y/o** | 521(20.7%) | 226(26.7%) | <0.001 | 256(31.1%) | 208(25.3%) | 0.010 |  |
| ***Subtype Identification*** |  |  |  |  |  |  |  |
| **CT** | 1481(58.9%) | 539(63.7%) | 0.017 | 529(64.4%) | 520(63.3%) | 0.692 |  |
| **MRI** | 808(32.1%) | 283(33.5%) | 0.391 | 267(32.5%) | 278(33.8%) | 0.289 |  |
| **AVS** | 471(18.7%) | 190(22.5%) | 0.023 | 151(18.4%) | 195(23.7%) | 0.020 |  |
| **NP-59** | 304(12.1%) | 113(13.4%) | 0.186 | 99(12.0%) | 129(12.9%) | 0.343 |  |
| **Posture** | 1085(43.1%) | 413(48.8%) | 0.336 | 379(46.1%) | 399(48.5%) | 0.421 |  |

**Abbreviations:** AVS, adrenal venous sampling, CT, computer tomography, MRI, Magnetic Resonance Imaging, NP-59, 131I-6β-Iodomethyl-19-Norcholesterol SPECT/CT

**Table S2.** Comparison of characteristics between survivors and non-survivors in PA patients.

|  | **Survivor (n=2910)** | **Non-survivor (n=452)** | ***p*** |
| --- | --- | --- | --- |
| **Male gender** | 1300 (44.7%) | 257 (56.9%) | <0.001 |
| **Age (in year)** | 49.6 ± 13.7 | 62.7 ± 15.7 | <0.001 |
| ***Premorbid risk*** | | | |
| **Congestive heart failure** | 155 (5.3%) | 102 (22.6%) | <0.001 |
| **Cerebrovascular disease** | 322 (11.1%) | 121 (26.8%) | <0.001 |
| **CKD** | 162 (5.6%) | 77 (17.0%) | <0.001 |
| **COPD** | 241 (8.3%) | 114 (25.2%) | <0.001 |
| **Coronary artery disease** | 41(1.4%) | 31(6.9%) | <0.001 |
| **Dementia** | 29 (1.0%) | 30 (6.6%) | <0.001 |
| **Diabetes Mellitus** | 569 (19.6%) | 169 (37.4%) | <0.001 |
| **Gout** | 261 (9.0%) | 55 (12.2%) | <0.001 |
| **Hemiplegia** | 18 (0.6%) | 19 (4.2%) | <0.001 |
| **Hyperlipidemia** | 619 (21.3%) | 87 (19.2%) | <0.001 |
| **Hypokalemia** | 1318 (45.3%) | 250 (55.3%) | <0.001 |
| **Moderate or Severe liver disease** | 227 (7.8%) | 77 (17.0%) | <0.001 |
| **Peptic Ulcer** | 487 (16.7%) | 137 (30.3%) | <0.001 |
| **Peripheral vascular disease** | 27 (0.9%) | 11 (2.4%) | 0.013 |
| **Rheumatologic disease** | 40 (1.4%) | 4 (0.9%) | 0.508 |
| **Solid tumor** | 126 (4.3%) | 55 (12.2%) | <0.001 |
| ***Antihypertensive drugs used at diagnosis*** | | | |
| **α- blockers** | 374(12.8%) | 65(14.3%) | 0.368 |
| **β- blockers** | 1084(37.3%) | 173(38.3%) | 0.676 |
| **Calcium channel blockers** | 1756(60.3%) | 298(65.9%) | 0.026 |
| **Diuretics** | 507(17.4%) | 152(33.6%) | <0.001 |
| **ACEI/ ARB** | 1152 (39.6%) | 213 (47.1%) | 0.003 |
| ***Treatment after PA diagnosis*** | | | |
| **Adrenalectomy** | 814 (28.0%) | 32(7.1%) | <0.001 |
| **K supply** | 1229 (42.2%) | 287 (63.5%) | <0.001 |
| **Median cumulative dose of MRA (expressed as DDD*)** | 0.05 ± 0.21 | 0.07 ± 0.21 | 0.198 |
| ***Outcomes*** |  |  |  |
| **Cardiovascular events** | 560 (19.2%) | 190 (42.0%) | <0.001 |

**Table S3.** Factors identified as predictors of adrenalectomy after PA diagnosis in the logistic regression model of propensity score.

| **Factor** | **OR** | **Lower 95% CI** | **Upper**  **95% CI** | ***P*** |
| --- | --- | --- | --- | --- |
| **Age** | 0.97 | 0.96 | 0.98 | <0.001 |
| **Male gender** | 0.87 | 0.73 | 1.02 | 0.087 |
| ***Baseline comorbidities*** |  |  |  |  |
| **CKD** | 0.50 | 0.33 | 0.75 | <0.001 |
| **Congestive heart failure** | 0.69 | 0.45 | 1.03 | 0.075 |
| **COPD** | 0.61 | 0.43 | 0.86 | <0.001 |
| **Dementia** | 0.21 | 0.04 | 0.71 | 0.026 |
| **Hyperlipidemia** | 0.68 | 0.54 | 0.84 | <0.001 |
| **Hypokalemia** | 1.58 | 1.34 | 1.87 | <0.001 |
| **Moderate or severe liver disease** | 0.73 | 0.53 | 0.98 | 0.043 |

Hosmer-Lemeshow goodness of fit (GOF) test P =0.651; AUC (area under curve) = 0.703.

This logistic regression model was adjusted with age, gender, congestive heart, failure, cerebrovascular disease, CKD, COPD, coronary artery disease, dementia, diabetes mellitus, gout, hemiplegia, hyperlipidemia, hypokalemia, K supply, moderate or severe liver disease, peptic ulcer, peripheral artery disease, rheumatologic disease and tumor.

**Abbreviations:** ACEI, angiotensin converting enzyme inhibitor; ARB, angiotensin receptor blocker; CI, confidence interval; CKD, chronic kidney disease; COPD, chronic obstructive pulmonary disease; OR, odds ratio; PA, primary aldosteronism.

**Table S4.** Factors associated with long-term mortality in the Cox regression model* taking into account the daily dose of MRA.

|  | | **HR** | **Lower**  **95% CI** | **Upper**  **95% CI** | ***p*** |
| --- | --- | --- | --- | --- | --- |
| **Age (in year)** | 1.05 | | 1.04 | 1.06 | <0.001 |
| **Male gender** | 1.28 | | 1.06 | 1.55 | 0.011 |
| **Premorbid risk** | | | | |  |
| **CKD** | 2.10 | | 1.61 | 2.73 | <0.001 |
| **Congestive heart failure** | 1.71 | | 1.34 | 2.17 | <0.001 |
| **COPD** | 1.87 | | 1.02 | 3.43 | 0.044 |
| **Coronary artery disease** | 1.99 | | 1.36 | 2.92 | <0.001 |
| **Dementia** | 1.53 | | 1.03 | 2.27 | 0.034 |
| **Diabetes Mellitus** | 1.55 | | 1.27 | 1.90 | <0.001 |
| **Solid tumor** | 1.78 | | 1.33 | 2.40 | <0.001 |
| **Time-varying covariate** | | | | | |
| **Adrenalectomy** | 0.26 | | 0.18 | 0.38 | <0.001 |
| **K supply** | 2.32 | | 1.89 | 2.85 | <0.001 |
| **Daily dose of MRA (expressed as DDD)** | | | | | |
| **DDD< 0.17 v.s. [0.17-0.66]** | 1.27 | | 1.03 | 1.57 | 0.029 |
| **DDD> 0.66 v.s. [0.17-0.66]** | 3.05 | | 1.23 | 7.55 | 0.016 |

* The final model had a good validity (C-index =0.83).

The model adjusted for age, gender, congestive heart failure, cerebrovascular disease, CKD, COPD, coronary artery disease, DDD, dementia, diabetes mellitus, gout, hemiplegia, hyperlipidemia, hypokalemia, K supply, moderate or severe liver disease, peptic ulcer , peripheral artery disease, rheumatologic disease, and tumor.

****** Each patient ’ s exposure to MRA (belonging to the ATC class C03D) was measured on the basis of the cumulative dose, and expressed as the defined daily dose (DDD) according to the definition by World Health Organization. The DDD of MRA was calculated form 30 days to 365 days before mortality or the end of the study (December 31, 2010), whichever occurred first.

**Abbreviations:** CI, confidence interval; CKD, chronic kidney disease; COPD, chronic obstructive pulmonary disease; DDD, defined daily dose; HR, hazard ratio; K, potassium; MRA, mineralocorticoid receptor antagonist.

Supplementary Fig S1.

Kaplan-Meier curves of freedom from mortality for PA patients receiving adrenalectomy, and those without operation.

(Abbreviation: PA, primary aldosteronism)


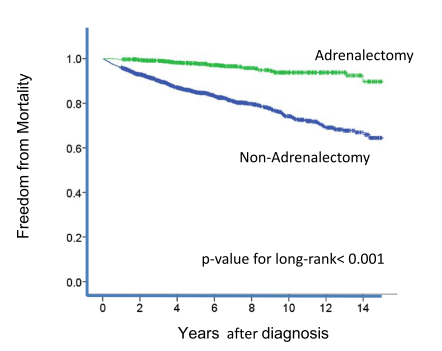


1. Wu, V.C.*, et al.* Primary aldosteronism: diagnostic accuracy of the losartan and captopril tests. *Am J Hypertens* **22**, 821-827 (2009).

2. Yen, R.F.*, et al.* 131I-6beta-iodomethyl-19-norcholesterol SPECT/CT for primary aldosteronism patients with inconclusive adrenal venous sampling and CT results. *J Nucl Med* **50**, 1631-1637 (2009).

3. Lu, C.C.*, et al.* Prognostic value of semiquantification NP-59 SPECT/CT in primary aldosteronism patients after adrenalectomy. *Eur J Nucl Med Mol Imaging* **41**, 1375-1384 (2014).

4. Lee, B.C.*, et al.* Evaluation of right adrenal vein anatomy by Dyna computed tomography in patients with primary aldosteronism. *Scientific reports* **6**, 28305 (2016).

5. Wu, V.C.*, et al.* Administrative data on diagnosis and mineralocorticoid receptor antagonist prescription identified patients with primary aldosteronism in Taiwan. *Journal of clinical epidemiology* **67**, 1139-1149 (2014).

6. Wu, V.C.*, et al.* Endothelial Progenitor Cells in Primary Aldosteronism: A Biomarker of Severity for Aldosterone Vasculopathy and Prognosis. *J Clin Endocrinol Metab* **96**, 3175-3183 (2011).
